# RNA-induced liquid phase separation of SARS-CoV-2 nucleocapsid protein facilitates NF-κB hyper-activation and inflammation

**DOI:** 10.1038/s41392-021-00575-7

**Published:** 2021-04-24

**Authors:** Yaoxing Wu, Ling Ma, Sihui Cai, Zhen Zhuang, Zhiyao Zhao, Shouheng Jin, Weihong Xie, Lingli Zhou, Lei Zhang, Jincun Zhao, Jun Cui

**Affiliations:** 1 MOE Key Laboratory of Gene Function and Regulation, State Key Laboratory of Biocontrol, School of Life Sciences, Sun Yat-sen University, Guangzhou, Guangdong China; 2 State Key Laboratory of Respiratory Disease, Guangzhou Institute of Respiratory Disease, The First Affiliated Hospital of Guangzhou Medical University, Guangzhou, Guangdong China

**Keywords:** Infection, Inflammation, Innate immunity

## Abstract

The ongoing 2019 novel coronavirus disease (COVID-19) caused by SARS-CoV-2 has posed a worldwide pandemic and a major global public health threat. The severity and mortality of COVID-19 are associated with virus-induced dysfunctional inflammatory responses and cytokine storms. However, the interplay between host inflammatory responses and SARS-CoV-2 infection remains largely unknown. Here, we demonstrate that SARS-CoV-2 nucleocapsid (N) protein, the major structural protein of the virion, promotes the virus-triggered activation of NF-κB signaling. After binding to viral RNA, N protein robustly undergoes liquid–liquid phase separation (LLPS), which recruits TAK1 and IKK complex, the key kinases of NF-κB signaling, to enhance NF-κB activation. Moreover, 1,6-hexanediol, the inhibitor of LLPS, can attenuate the phase separation of N protein and restrict its regulatory functions in NF-κB activation. These results suggest that LLPS of N protein provides a platform to induce NF-κB hyper-activation, which could be a potential therapeutic target against COVID-19 severe pneumonia.

## Introduction

At present, the ongoing 2019 novel coronavirus disease (COVID-19) caused by severe acute respiratory syndrome coronavirus 2 (SARS-CoV-2/2019-nCoV) has turned into a worldwide pandemic with over 100 million confirmed cases and tolled at 2.5 million deaths. Daily new registered cases indicate the continuously sharp growth in many countries and regions, but the targeted SARS-CoV-2 therapy development is hampered due to the deficiency of multi-aspect understanding, including the pathogen biology, immune evasion, and pathogenesis.^1^ Therefore, for novel therapeutic interventions, an urgent task was proposed to broaden the acquaintance on host–pathogen biology of SARS-CoV-2.

Innate and adaptive immune responses on SARS-CoV-2 infection generally begin at cellular level after viral evasion.^2–4^ SARS-CoV-2 has a preferential tropism toward airway epithelial cells through approaching to the angiotensin-converting enzyme 2 (ACE2) receptor.^5–7^ During infection, SARS-CoV-2 attacks and replicates in human airway epithelial cells, which triggers the activation of inflammatory responses and leads to subsequent virus-triggered pyroptosis and release of inflammatory cytokines.^1,8^ Pattern recognition receptors (PRRs) of alveolar epithelial cells and alveolar macrophages are then activated by targeting a set of pathogen-associated molecular patterns (PAMPs) or damage-associated molecular patterns (DAMPs).^1,9^ PRRs then activate the inflammatory signaling pathway, triggering a wave of local inflammation and a robust secretion of pro-inflammatory cytokines and chemokines, including IL-6 and IFNγ.^8^ Cytokines and chemokines secretion attract more immune cells to the infected site, particularly monocytes and T cells, as the infiltration of lymphocytes into airways could be observed at about 80% patients with SARS-CoV-2 infection.^10,11^ In most cases, recruited immune cells remove SARS-CoV-2 in the lung and reduce the immune responses. However, in some individuals, the immune responses become dysfunctional, accompanied by cytokine storms and widespread lung inflammation. For instance, IL-6 levels grow continuously over time in severe patients of COVID-19 and are more elevated in non-survivors than survivors.^12^ Thus, the disease severity or mortality of COVID-19 are linked with dysfunctional inflammatory responses and cytokine storms.^1,9^

The expression of IL-6 is controlled by a variety of transcription factors with consensus sequences at its promoter region.^13,14^ Nuclear factor κB (NF-κB) is one of the key transcription factors of IL-6 in both immune cells and non-immune cells.^13,14^ Upon sensing different ligands such as tumor necrosis factor alpha (TNF-α), IL-1β, viruses, bacteria, or mitogens, their receptors then recruit the adaptor proteins, including myeloid differentiation primary response gene 88 (MyD88), receptor-interacting protein (RIP1), or TIR-domain-containing adapter-inducing interferon-β (TRIF).^15^ These adaptors promote the activation and self-ubiquitination of tumor necrosis factor receptor (TNF-R)-associated factor (TRAF) signaling molecules, thus recruiting TGF-beta-activated kinase 1 (TAK1) and IκB kinase (IKK) complex.^16^ Activated IKK complex then phosphorylates IκB proteins and leads to its ubiquitin-proteasome degradation. Degradation of IκB allows NF-κB translocation to the nucleus to initiate the transcription of downstream genes by binding to specific DNA binding regions.^17^ Recent single-cell sequencing and RNA-sequencing researches showed that SARS-CoV-2 infection was capable of inducing NF-κB activation in peripheral blood mononuclear cells or bronchoalveolar immune cells from COVID-19 patients or in human respiratory cell lines.^3,18–21^ However, the mechanisms by which SARS-CoV-2 induces NF-κB activation are yet to be investigated.

SARS-CoV-2 is a member of coronaviridae family, which are enveloped viruses containing the largest genome (about 30,000 nucleotides in length) among positive-sense, single-stranded RNA viruses. The genomes of coronaviruses encode 14 major open reading frames (ORFs), which can be processed into four structural proteins, including spike (S), membrane (M), envelope (E) and nucleocapsid (N) proteins, 16 nonstructural proteins (nsp1 to 16) and at least 8 accessory proteins. Among these viral proteins, N protein is the central component of virions, which restructures viral RNA into ribonucleoprotein complex (vRNPs) and initiates viral assembly.^22,23^ Here, we demonstrate that SARS-CoV-2 N protein promotes the activation of NF-κB signaling by enhancing the association between TAK1 and IKK complex. By associating with viral RNA, N protein of SARS-CoV-2 undergoes liquid–liquid phase separation (LLPS), and forms functional membrane-less organelles to recruit TAK1 and IKK complex, thus promoting NF-κB activation. Consistently, 1.6-hexanediol, the inhibitor of LLPS, attenuates SARS-CoV-2-induced NF-κB activation. Our findings reveal that LLPS of N protein/RNA contributes to the virus-triggered inflammatory responses, which may shed light on developing novel strategies and potential therapeutic options for COVID-19.

## Results

### Defining the inflammatory responses to SARS-CoV-2

**Fig. 1:**
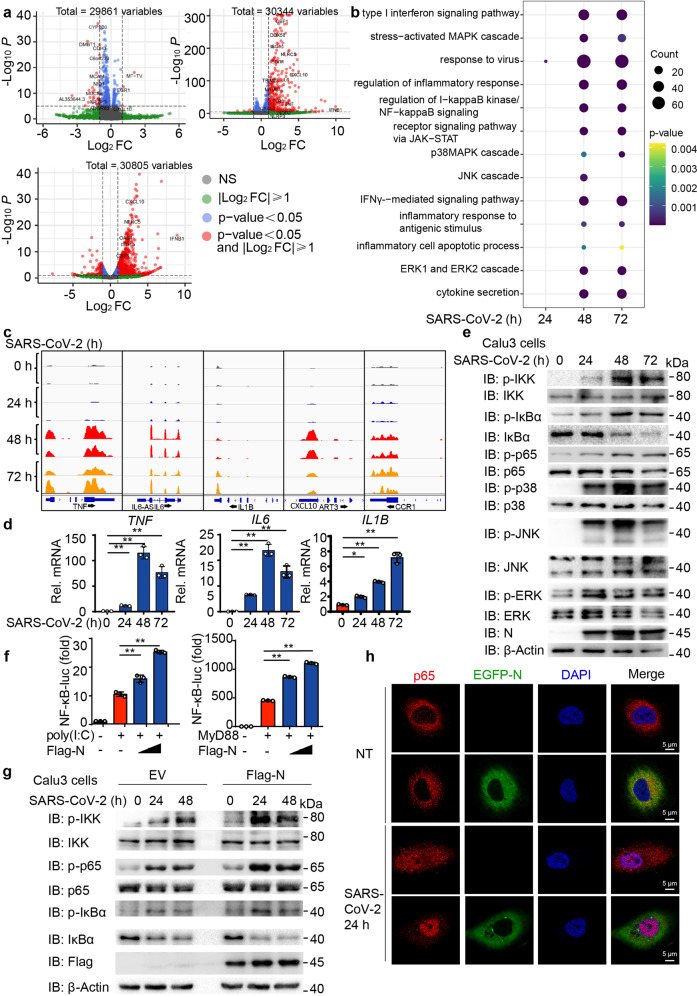
N protein of SARS-CoV-2 enhances SARS-CoV-2-triggered NF-κB signaling. **a** Volcano plot of DEGs comparing SARS-CoV-2 infection cells versus negative control (NC). Calu3 cells were infected SARS-CoV-2 at MOI = 0.05 for 24, 48, and 72 h. RNA-seq was performed on poly(A)-enriched total RNA. ns not significant. **b** Dot-plot visualization of enriched GO terms showing the upregulated genes from (**a**). The color of the dots represents the *p*-value for each enriched GO term, and the size represents the reads count of genes enriched in the total gene set. **c** IGV browser tracks showing the RNA-seq signals of inflammatory response genes in Calu3 cells at indicated time points post infection. **d** Quantitative PCR with reverse transcription analysis of NF-κB relative cytokines genes (*TNF*, *IL6*, *IL1B*) of Calu3 cells with SARS-CoV-2 infection at MOI = 0.05 for indicated time points. **e** Calu3 cells were infected with SARS-CoV-2 at MOI = 0.05 for indicated time points and the lysates were harvested for immunoblot analysis with indicated antibodies. **f** HEK293T cells were transfected with NF-κB-luciferase (luc) reporter along with Flag- empty vector (EV) or Flag-N protein, followed by transfecting plasmid expressing MyD88 or 1 μg/mL poly(I:C) treatment for 12 h. The lysates were collected 36 h post-transfection and luciferase activities were tested. **g** Calu3 cells stably expressing N protein were infected by SARS-CoV-2 at MOI = 0.05 for indicated time points and cell lysates were harvested for immunoblot analysis with indicated antibodies. **h** Huh7 cells stably expressing EGFP-N protein were infected by SARS-CoV-2 at MOI = 0.2 for 24 h or left non-treated (NT), followed by labeling p65 and DAPI. Confocal microscopy of p65 localization was performed. Scale bars, 5 μm. Data in (**d**) and (**f**) are expressed as mean ± SEM of at least three independent experiments. **p < 0.01, ns not significant (two-tailed Student’s *t*-test). For (**e**) (**g**) and (**h**) similar results are obtained for three independent biological experiments

To investigate the host transcriptional landscape in response to SARS-CoV-2, we treated Calu3 (a human respiratory cell line) cells with SARS-CoV-2 (Accession number: MT123290) for 24, 48 or 72 h at an MOI of 0.05 and collected the poly-A enriched RNA from the SARS-CoV-2-infected Calu3 cells to perform the global RNA-sequencing analysis (RNA-seq). Analysis of genes expressed differentially were determined by comparing SARS-CoV-2-infected cells transcriptomes with non-infected control group. The cut-off of fold change (FC) ratio (|log2FC| ≥ 1) and adjusted *P* value (*p* value < 0.05) were used to identify differentially expressed genes (DEGs). The volcano plot depicting the amount of DEGs in different time point post infection revealed that a few of genes were down-regulated at the early time point of 24 h post infection and a large number of genes were significantly upregulated with SARS-CoV-2 infection for 48 or 72 h (Fig. 1a). The gene-ontology enrichment analyses illustrated that “type I interferon (IFN) signaling pathway” ranked first in the upregulated pathways in response to SARS-CoV-2-infection. In addition to robust IFN responses, SARS-CoV-2 infection in Calu3 cells also elicited a “regulation of inflammatory response”, along with up-regulation of “regulation of I-κB kinase/NF-κB signaling” and “cytokine secretion” pathways (Fig. 1b). To confirm the pathway enrichment during SARS-CoV-2 infection, we performed gene-set-enrichment analyses (GSEA) at 48 h post infection.^24^ Consistently, GSEA analysis showed enrichment (FDR < 0.01) for signatures associated with type I IFN signaling pathway and NF-κB signaling (Supplementary Fig. S1a). The heatmap of top 100 DEGs also revealed that principal upregulated genes and associated signatures with SARS-CoV-2 infection were corresponding to type I IFN antiviral responses and inflammatory responses (*TNF*, *IL6*, *IL1B*, *CXCL10*, *CXCL8*, et al.), which were also identified by IGV genome browser directly (Fig. 1c and Supplementary Fig. S1b, c). RNA-expression analysis by real-time quantitative PCR (RT-qPCR) of these upregulated genes further confirmed the effects of SARS-CoV-2 infection on genes encoding products associated with inflammatory responses (*TNF*, *IL6*, *IL1B*, *CXCL10*, *CXCL8*, and *CCR1*) (Fig. 1d and Supplementary Fig. S1d). To further investigate SARS-CoV-2-induced inflammatory responses, we detected the effect of SARS-CoV-2 infection on mitogen-activated protein kinase (MAPK) signal transduction pathway and nuclear factor-κB/inhibitory factor-κB (NF-κB/IκB) signal pathway. Immunoblot analysis showed that SARS-CoV-2 infection induced the activation of MAPK pathway and NF-κB pathway, and enhanced the phosphorylation level of p38, JNK, ERK, IKK, IκBα, and p65, thus leading to the degradation of IκBα in Calu3 cells and Huh7 cells, a human hepatoma cell line permissive to SARS-CoV-2 infection (Fig. 1e and Supplementary Fig. S2a). The reduction of the protein level of several proteins such as p65 at 72 h might be due to SARS-CoV-2-triggered cell death.^1,25–27^ Consistent with the previous studies,^2,3,18,20,21^ our results revealed that SARS-CoV-2 infection could lead to the activation of host inflammatory responses, including NF-κB signaling.

### Nucleocapsid protein of SARS-CoV-2 enhances virus-triggered NF-κB signaling

As a hallmark of most infections, NF-κB serves as a potential therapeutic target for infectious diseases. Inhibitors that specifically target NF-κB pathway,^28–34^ reduced the inflammatory responses in SARS-CoV-2-infected Calu3 cells (Supplementary Fig. S2b), which is consistent with previous studies on SARS-CoV infection.^9,35,36^ These results indicated the pathogenic roles of NF-κB activation during SARS-CoV-2 infection. To discover the participation of SARS-CoV-2 in activating NF-κB pathway, we next ectopically expressed different proteins of SARS-CoV-2 and studied their functions in NF-κB signaling pathway. 293T cells were transfected with empty vector (EV) plasmid or with plasmids expressing SARS-CoV-2 proteins, together with luciferase reporter plasmids containing NF-κB promoter (NF-κB-luc) and a control luciferase plasmid (TK-luc), followed with poly(I:C) treatment or transfection of MyD88. We found that proteins of SARS-CoV-2 exhibited divergent effects on NF-κB activation, whereas nucleocapsid protein (N) of SARS-CoV-2 exerted a positive role in poly(I:C)-/MyD88-mediated activation of NF-κB pathway (Supplementary Fig. S2c, d). Subsequent analysis showed that SARS-CoV-2 N protein promoted NF-κB activation with poly(I:C) treatment and MyD88 transfection in a dose-dependent manner (Fig. 1f). In order to confirm the cellular functions of SARS-CoV-2 N protein in NF-κB pathway, we generated SARS-CoV-2 N-stably-expressed Calu3 and Huh7 cells, and found that N protein markedly enhanced the phosphorylation level of IKK, p65, and IκBα, but not the phosphorylation of p38, ERK, and JNK after SARS-CoV-2 infection or poly(I:C) and TNFα treatment (Fig. 1g and Supplementary Fig. S3a). Consistently, overexpression of SARS-CoV-2 N protein promoted nuclear translocation of p65 after SARS-CoV-2 infection or poly(I:C)/TNFα combination treatment (Fig. 1h and Supplementary Fig. S3b–d). SARS-CoV-2 N protein could also enhance the virus-triggered induction of NF-κB downstream responsive pro-inflammatory cytokines, including *TNF*, *IL6*, and *CXCL10* (Supplementary Fig. S3e). Collectively, these results indicated that SARS-CoV-2 N protein functioned as a positive regulator in virus-triggered NF-κB signaling.

### SARS-CoV-2 N protein promotes NF-κB signaling at TAK1 and IKK level

**Fig. 2:**
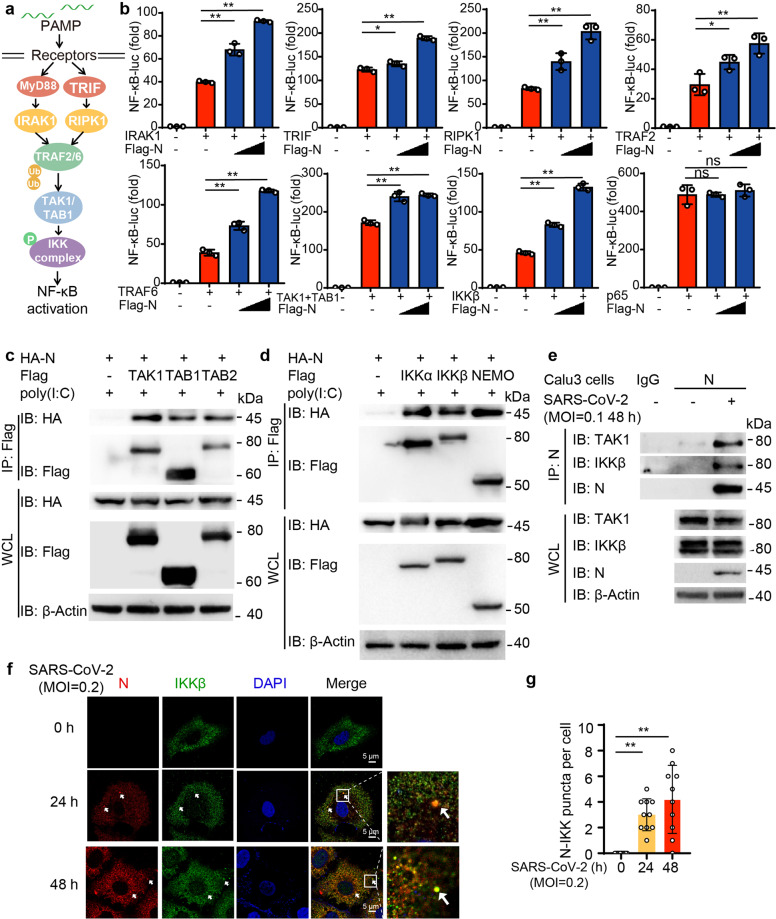
SARS-CoV-2 N protein promotes NF-κB signaling at TAK1 and IKK complex level. **a** Schematic overview of NF-κB signaling pathway. **b** HEK293T cells were transiently transfected with NF-κB luciferase reporter, Flag-empty vector (EV) and increasing amount of Flag-N protein together with plasmids expressing IRAK1, TRIF, RIPK1, TRAF2, TRAF6, TAK1 and TAB1, IKKβ and p65. Cell lysates were collected at 36 h post-transfection and luciferase activities were tested. **c** HEK293T cells were transiently transfected with HA-N protein plasmid along with Flag-empty vector (EV), Flag-TAK1, Flag-TAB1, and Flag-TAB2 plasmids, followed with 1 μg/mL poly(I:C) treatment for 6 h before harvested. Whole-cell lysates were subjected to immunoprecipitation with anti-Flag beads and immunoblot analysis was performed with indicated antibodies. **d** HEK293T cells were transiently transfected with HA-N protein plasmid along with Flag-EV, Flag-IKKα, Flag-IKKβ, and Flag-NEMO plasmids, followed with 1 μg/mL poly(I:C) treatment for 6 h before harvested. Whole-cell lysates were subjected to immunoprecipitation with anti-Flag beads and immunoblot analysis was performed with indicated antibodies. **e** Extracts of Calu3 cells infected with SARS-CoV-2 at MOI = 0.1 for 48 h were subjected to immunoprecipitation with anti-SARS-CoV-2 N protein and immunoblot analysis with indicated antibodies. **f**–**g** Representative images (**f**) of confocal microscopy of co-localization between SARS-CoV-2 N protein and IKKβ in Huh7 cells with SARS-CoV-2 infection at MOI = 0.2 for indicated time points followed by labeling of N protein, IKKβ, and DAPI. Scale bars, 5 μm. Quantitative analysis (**g**) of the puncta between IKK and SARS-CoV-2 N protein (10 cells per sample). Data in (**b**) are expressed as mean ± SEM of at least three independent experiments. Data in (**g**) are expressed as mean ± SD. *p < 0.05, **p < 0.01, ns not significant (two-tailed Student’s *t*-test). For (**c**–**f**), similar results are obtained for three independent biological experiments

We next sought to determine how SARS-CoV-2 N protein promotes NF-κB activation. We co-transfected N protein along with key molecules in NF-κB pathway and detected the activation of NF-κB signaling. As shown in Fig. 2a, b, overexpression of SARS-CoV-2 N protein improved the activation of NF-κB induced by IRAK1, TRIF, RIPK1, TRAF2, TRAF6, TAK1/TAB1 or IKKβ, but not p65. These data indicated that SARS-CoV-2 N protein promoted NF-κB activation by targeting IKK complex. We next investigated the association between N proteins and the kinases in NF-κB signaling. Co-immunoprecipitation analysis revealed that SARS-CoV-2 N protein interacted with TAK1 complex and IKK complex, including TAK1, TAB1, TAB2, IKKα, IKKβ, and NEMO (Fig. 2c, d). We further detected the association between N protein and TAK1 or IKKβ, and found that poly(I:C) treatment enhanced their interactions (Supplementary Fig. S3f, g). Immunoprecipitation analysis also showed that SARS-CoV-2 N protein could endogenously associate with IKK and TAK1 during infection (Fig. 2e). Immunofluorescence analysis further revealed the enhanced co-localization between N protein and IKKβ after SARS-CoV-2 infection in Huh7 cells (Fig. 2f, g). Overall, these results suggested that SARS-CoV-2 N protein targets TAK1 and IKK complex to increase NF-κB activation.

### SARS-CoV-2 N protein undergoes LLPS to promote NF-κB signaling

**Fig. 3:**
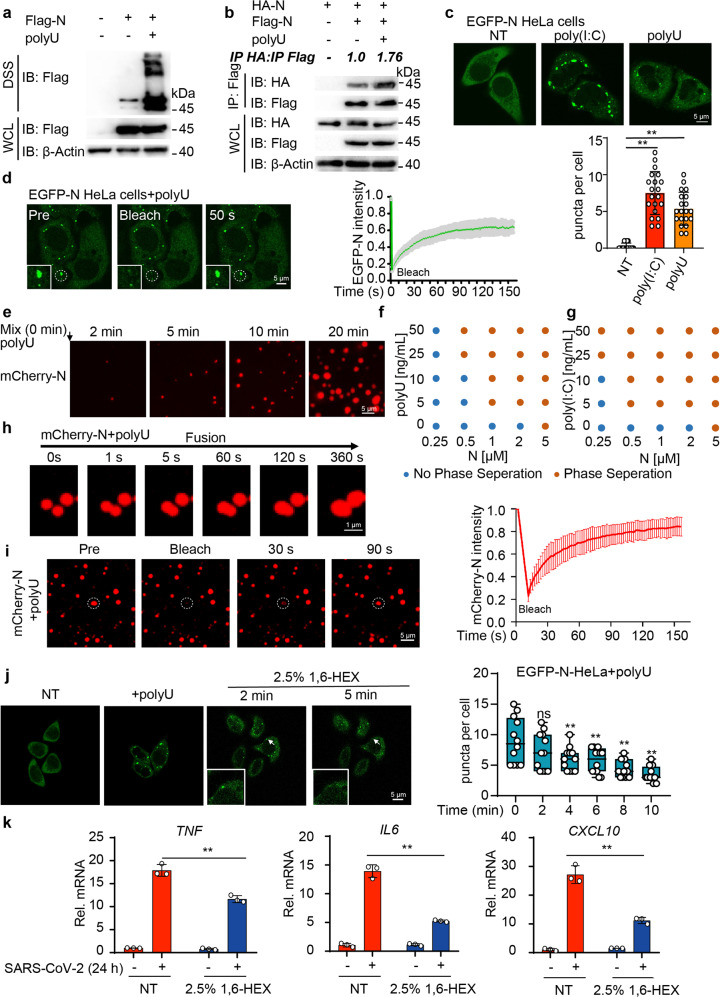
SARS-CoV-2 N protein undergoes LLPS to promote NF-κB signaling. **a** HEK293T cells were transfected with Flag-empty vector (EV) and Flag-N plasmid, treated with 1 μg/mL polyU for 6 h. Cell lysates were collected and treated with DSS for immunoblotting with indicated antibodies. **b** HEK293T cells were transfected with HA-N along with Flag-EV and Flag-N plasmids, treated with 1 μg/mL polyU for 6 h. Whole-cell lysates were subjected to immunoprecipitation with anti-Flag beads and immunoblot analysis was performed with indicated antibodies. The ratio of the gray value between IP-HA and IP-Flag was determined. **c** Representative live-cell images (up) of HeLa cells stably expressing EGFP-N protein with 1 μg/mL poly(I:C) or 1 μg/mL polyU treatment for 6 h or left non-treated (NT). Scale bars, 5 μm. Quantitative analysis (down) of N protein droplets (20 cells each sample). **d** Representative images (left) of fluorescence recovery after photobleaching (FRAP) assay of N protein-polyU droplets in HeLa cells. HeLa cells stably expressing EGFP-N protein with 1 μg/mL polyU treatment for 6 h before bleaching. Bleaching was performed at the indicated time points and the recovery occurred at 37 °C. Scale bars, 5 μm. Fluorescence intensity analysis (right) of FRAP over a 150 s time course. **e** Representative images of time-lapse imaging of N protein-polyU phase separation in vitro. Liquid droplets were formed after mixing of 10 μM mCherry-N protein with 10 ng/mL polyU and matured at 37 °C for 20 min. Scale bars, 5 μm. **f**–**g** Phase separation diagram of mCherry-N protein with polyU (**f**) or poly(I:C) (**g**) at the indicated concentrations in vitro. **h** Representative images of fusion of two mCherry-N protein-polyU puncta in vitro. 10 μM mCherry-N protein was mixed with 10 ng/mL polyU at 37 °C. Scale bars, 1 μm. **i** Representative images (left) of the FRAP assay of N protein-polyU droplets in vitro. 10 μM mCherry-N protein mixed with 10 ng/mL polyU and matured at 37 °C for 5 min. Bleaching was performed at the indicated time points and the recovery occurred at 37 °C. Scale bar, 5 μm. Fluorescence intensity analysis (right) of FRAP over a 150 s time course. **j** Time-lapse micrographs (left) of HeLa cells stably expressing EGFP-N protein with 1 μg/mL polyU treatment for 6 h, together with 20 μg/mL digitonin and 2.5% 1.6-HEX treatment for indicated time or left non-treated (NT). Scale bar, 5 μm. The quantitative analysis (right) of N protein droplets (12 cells each sample). **k** Quantitative PCR with reverse transcription analysis of NF-κB relative cytokines genes (*TNF*, *IL6*, *CXCL10*) of Calu3 cells with SARS-CoV-2 infection at MOI = 0.05 for 24 h followed by treatment of 20 μg/mL digitonin and 2.5% 1.6-HEX for 2 h. Data in (**d**), (**i**), and (**k**) are expressed as mean ± SEM of at least three independent experiments. Data in (**c**), (**j**) are expressed as mean ± SD. **p < 0.01, ns not significant (two-tailed Student’s *t*-test). For (**a**–**e**) and (**h**–**j**), similar results are obtained for three independent biological experiments

It has been reported that N protein of SARS-CoV and SARS-CoV-2 showed a high tendency to undergo oligomerization during infection.^23,37,38^ Disuccinimidyl suberate (DSS) treatment and immunoprecipitation assays revealed that both poly(I:C) and polyU enhanced homo-oligomerization of SARS-CoV-2 N protein (Fig. 3a, b and Supplementary Fig. S4a, b), suggesting viral RNA was essential for N protein oligomerization. Recent reports showed that SARS-CoV-2 also underwent LLPS in vitro.^39–43^ To investigate whether SARS-CoV-2 N protein forms liquid-like droplets in cells, we constructed HeLa and Huh7 cells stably expressing EGFP-labeled N protein, and found that N protein formed foci after poly(I:C) or polyU treatment through live-cell image (Fig. 3c). Fluorescence recovery experiments revealed that the fluorescence of poly(I:C)- or polyU-induced EGFP-N puncta recovered within 1 min after photobleaching (FRAP) (Fig. 3d and Supplementary Fig. S4c). The N protein/RNA puncta exhibited liquid-like properties, as fusion occurred between two puncta (Supplementary Fig. S4d). We further found that recombinant mCherry-fusion SARS-CoV-2 N protein formed micrometer-sized liquid droplets within 1 min when blended with poly(I:C) or polyU in vitro (Fig. 3e and Supplementary Fig. S4e). We next established the phase diagrams of N protein by performing the droplet formation assay with increasing concentrations of N protein and viral nucleic acid analogues. N protein was able to form liquid-like droplets at the concentration of 1 μM with the presence of poly(I:C) or polyU over 5 ng/mL (Fig. 3f, g). Small liquid droplets tended to coalesce and formed larger droplets over time, together with larger equivalent diameter in vitro (Fig. 3h). After bleaching, the fluorescence of mCherry-N droplets was efficiently recovered (Fig. 3i and Supplementary Fig. S4f). In addition, 1,6-hexanediol (1,6-HEX), a chemical probe to disrupt LLPS,^44^ dispersed N protein droplets (Fig. 3j and Supplementary Fig. S4g).

Since RNA binding drove a strong phase transition of SARS-CoV-2 N protein to liquid-like droplets, we next explored whether LLPS of N protein was necessary for its regulatory roles in NF-κB signaling. 1,6-HEX treatment reduced the function of N protein in NF-κB activation, as well as the induction of NF-κB-responsive downstream cytokines (Supplementary Fig. S4h, i). During SARS-CoV-2 infection, 1,6-HEX treatment not only impaired SARS-CoV-2-induced NF-κB-related inflammatory responses (Fig. 3k), but also inhibited SARS-CoV-2 replication (Supplementary Fig. S4j). These results demonstrate that RNA binding to SARS-CoV-2 N protein induces a robust phase separation in vitro and in cells, and the regulatory function on NF-κB of N protein is related to its LLPS.

### LLPS of SARS-CoV-2 N protein recruits and activates TAK1 and IKK complex to promote NF-κB activation

**Fig. 4:**
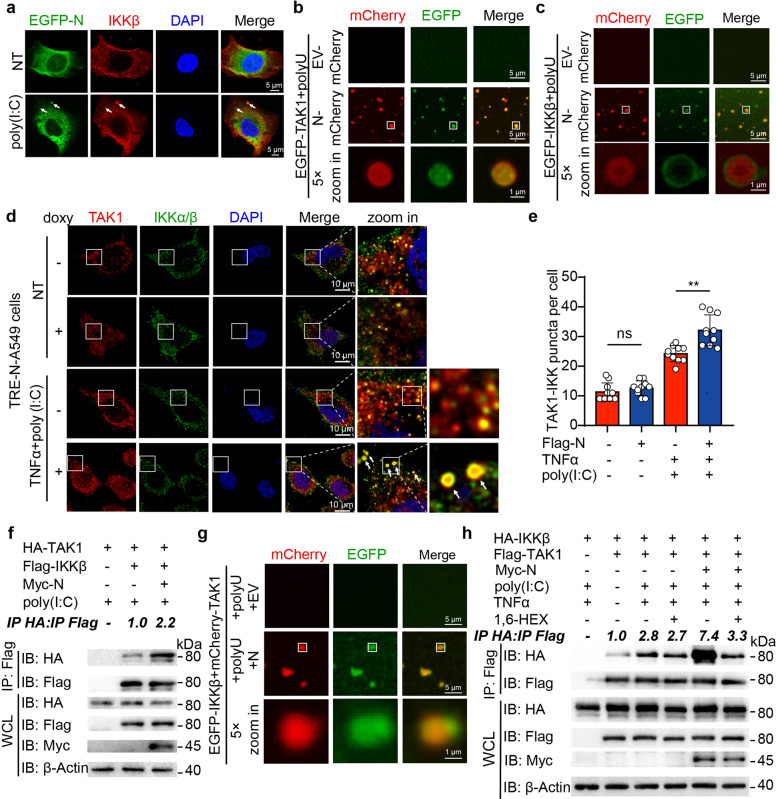
LLPS of SARS-CoV-2 N protein recruits TAK1 and IKK complex. **a** Representative image of confocal micrographs of Huh7 cells stably expressing EGFP-N protein, treated with 1 μg/mL poly(I:C) for 6 h, followed by labeling IKKβ and DAPI. Scale bars, 5 μm. **b**–**c** Representative images showing TAK1 (**b**) or IKKβ (**c**) were distributed into N protein-polyU droplets in vitro. 10 μM mCherry-N protein and 5 μM EGFP-TAK1 (**b**) or EGFP- IKKβ (**c**) were mixed with 10 ng/mL polyU and incubated at 37 °C for 10 min. Confocal 5× zoom-in of the area highlighted above was re-photographed. Scale bars, 5 μm. **d**–**e** Representative images (**d**) of confocal microscopy of TRE-N-A549 with 200 ng/mL doxy treated for 24 h or left untreated, followed by 1 µg/mL poly(I:C) treatment for 6 h or left non-treated (NT). Cells were fixed and labeled of TAK1 and IKKβ. Scale bars, 5 μm. Quantitative analysis (**e**) of the co-localization puncta (10 cells per sample). **f** HEK293T cells were transiently transfected HA-TAK1, Flag-IKKβ and Myc-N, followed by 1 µg/mL poly(I:C) treatment for 6 h. The whole-cell lysates were collected for immunoprecipitation by anti-Flag beads and immunoblotting by indicated antibodies. The ratio of the gray value between IP-HA and IP-Flag was determined. **g** Representative images of the formation of N protein-TAK1-IKKβ complex in vitro. 5 µM mCherry-TAK1 and EGFP-IKKβ were mixed with 10 ng/mL polyU and 10 μM His-N protein or 10 μM His-EV, then incubated at 37 °C for 10 min. Confocal 5× zoom-in of the area highlighted above was re-photographed. Scale bars, 5 μm. **h** HEK293T cells were transfected with HA-IKKβ, Flag-EV, Flag-TAK1, Myc-EV, and Myc-N protein with poly(I:C) and TNFα treatment, followed by 20 μg/mL digitonin and 2.5% 1.6-HEX for 2 h before harvested. Cell lysates were collected for immunoprecipitation with anti-Flag beads and immunoblotting with indicated antibodies. The ratio of the gray value between IP-HA and IP-Flag was determined. Data in (**e**) are expressed as mean ± SD. **p < 0.01, ns not significant (two-tailed Student’s *t*-test). For (**a**–**d**) and (**f**–**h**), similar results are obtained for three independent biological experiments

As LLPS is able to recruit additional components and promote protein–protein interaction or reactions, we investigated the mechanisms employed by SARS-CoV-2 N protein in regulating NF-κB activation through LLPS. As shown in Fig. 2c, d, N protein interacts with TAK1 and IKK complex, the key catalytic components in NF-κB cascade. Immunofluorescence assay showed that poly(I:C) and polyU increased the number of puncta between N protein and IKKβ, indicating the formation of IKK-N condensates (Fig. 4a). To find out whether LLPS of N protein recruits TAK1 and IKK complex to promote NF-κB cascade, we prepared recombinant EGFP-TAK1 and EGFP-IKKβ, and found that both TAK1 and IKKβ failed to form puncta with poly(I:C) or polyU alone, but they were shown to be recruited to the condensates of N protein in the presence of RNA in vitro (Fig. 4b, c and Supplementary Fig. S5a, b). In addition, N protein enhanced the co-localization between TAK1 and IKK complex after poly(I:C) and TNFα treatment in cells (Fig. 4d, e). Co-immunoprecipitation analysis revealed that SARS-CoV-2 N protein also facilitated the association between TAK1 and IKK complex (Fig. 4f and Supplementary Fig. S5c). Since K63-linked ubiquitin chains play an important role on the activation and recruitment of TAK1 and IKK complex, we performed ubiquitination assay and found that N protein significantly enhanced the K63-linked ubiquitin chains of TAK1 with poly(I:C) treatment (Supplementary Fig. S5d). Interestingly, recombinant mCherry-TAK1 and EGFP-IKKβ could form liquid-like droplet in the presence of SARS-CoV-2 N protein and polyU in vitro (Fig. 4g), suggesting N protein recruited IKK complex and TAK1 into liquid droplets. In addition, 1,6-HEX treatment reduced N protein-mediated TAK1/IKKβ association after poly(I:C) treatment (Fig. 4h), suggesting the regulatory roles of SARS-CoV-2 N protein in NF-κB signaling required its LLPS capacity. Collectively, these results suggest that SARS-CoV-2 N protein enhances the interaction between TAK1 and IKK complex by recruiting these components into N protein/RNA condensates.

### CTD domain of SARS-CoV-2 N protein is critical for its LLPS and NF-κB regulatory capability

**Fig. 5:**
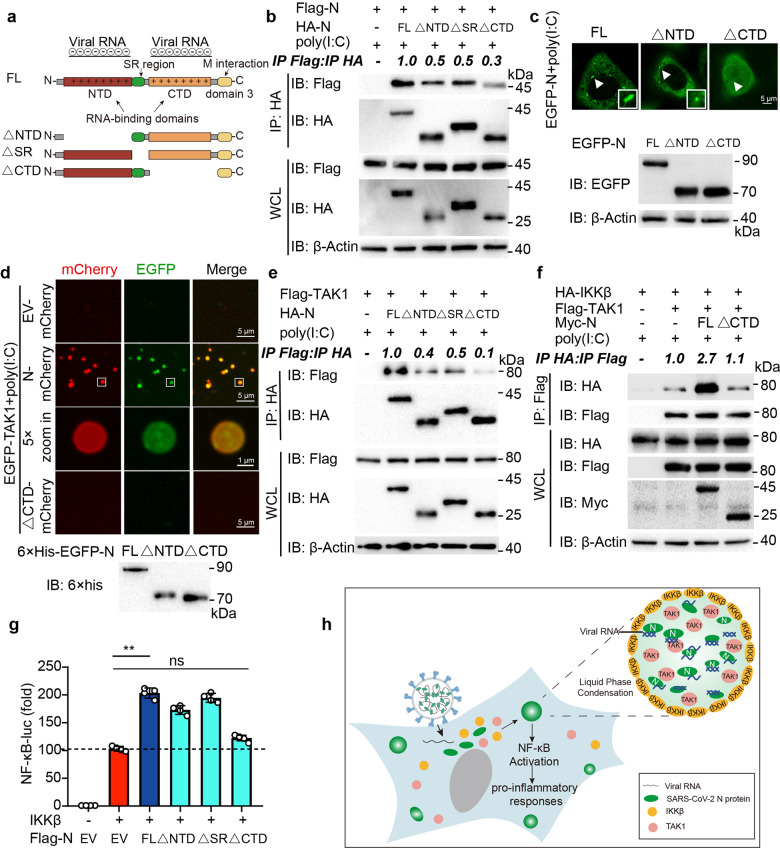
CTD domain of SARS-CoV-2 N protein CTD domain is critical for its LLPS and NF-κB regulatory capability. **a** Domain organization of SARS-CoV-2 N protein and its domain deletion mutants. **b** HEK293T cells were transfected Flag-N full-length (FL) along with HA-empty vector (EV), HA-N FL, △NTD, △SR, and △CTD. Whole-cell lysates were subjected to immunoprecipitation with anti-HA beads and immunoblot analysis was performed with indicated antibodies. The ratio of the gray value between IP-Flag and IP-HA was determined. **c** Representative images of live-cell confocal microscopy (up) of HeLa cells expressing EGFP-N FL, △NTD, and △CTD. Immunoblot analysis (down) were performed on the cell lysates with indicated antibodies. **d** Representative images showing TAK1 was distributed into N protein-poly(I:C) droplets in vitro. 10 µM mCherry-N protein or mCherry-N protein-△CTD, 5 µM EGFP-TAK1 were mixed with 10 ng/mL poly(I:C) and incubated at 37 °C for 10 min. Confocal 5× zoom-in of the area highlighted above was re-photographed. Scale bars, 5 μm. **e** Lysates of HEK293T cells transfected Flag-TAK1 and HA-EV, HA-N FL, △NTD, △SR, and △CTD, together with 1 µg/mL poly(I:C) for 6 h, were performed immunoprecipitation with anti-HA beads and immunoblotted by indicated antibodies. The ratio of the gray value between IP-Flag and IP-HA was determined. **f** Lysates of HEK293T cells transfected with HA-IKKβ and Flag-EV, Flag-TAK1, Myc-EV, Myc-N FL, and △CTD, followed by 1 µg/mL poly(I:C) treatment for 6 h were performed immunoprecipitation with anti-Flag beads and immunoblotted by indicated antibodies. The ratio of the gray value between IP-HA and IP-Flag was determined. **g** HEK293T cells were transiently transfected with NF-κB luciferase reporter, TK-luk, and IKKβ, together with Flag-EV, Flag-N FL, △NTD, △SR, and △CTD. Lysates were collected at 36 h post-transfection and luciferase activities were tested. **h** Schematic representation of SRAS-CoV-2 N protein regulating NF-κB signaling pathway by recruiting TAK1 and IKKβ through liquid–liquid phase separation. Data in (**g**) are expressed as mean ± SEM of at least three independent experiments. **p < 0.01, ns, not significant (two-tailed Student’s *t*-test). For (**b–f**), similar results are obtained for three independent biological experiments

To investigate the molecular basis of SARS-CoV-2 N protein underlying its function on NF-κB activation through LLPS, we constructed a panel of N protein mutants, including deletion of the N-terminal domain (△NTD), deletion of the C-terminal domain (△CTD) and deletion of the central a serine- and arginine-rich tract (△SR) based on a previous crystal and structural studies (Fig. 5a).^22^ Co-immunoprecipitation showed that △CTD mutant weakened its self-interaction (Fig. 5b). In addition, compared with full-length (FL) N protein and its △NTD mutant, △CTD mutant failed to form liquid-like puncta (Fig. 5c and Supplementary Fig. S6a, b). These results were consistent with previous findings that CTD domain of N protein was critical for the oligomerization of N protein.^22,39^ Intriguingly, △CTD mutant failed to form liquid droplets with TAK1 or IKKβ (Fig. 5d and Supplementary Fig. S6c), suggesting that CTD domain is related to the function on LLPS of N protein as well as the recruitment of TAK1/IKK. As expected, △CTD mutant showed a weak association with TAK1 and IKKβ (Fig. 5e and Supplementary Fig. S6d). Co-immunoprecipitation and immunofluorescence assays further confirmed that △CTD mutant failed to enhance the association between TAK1 and IKKβ (Fig. 5f and Supplementary Fig. S6e). In addition, N protein △CTD mutant exhibited a weaker regulatory capability on NF-κB signaling (Fig. 5g). Together, these results reveal that CTD domain of N protein plays an indispensable role in LLPS of N protein and its NF-κB regulatory capability.

## Discussion

Nowadays, COVID-19 caused by SARS-CoV-2 has rapidly spread over the world and become an ongoing global pandemic. Typical clinical symptoms of COVID-19 are fever, cough, myalgia, and difficulty in breathing,^8,11,45^ while severe patients develop acute respiratory distress syndrome (ARDS) and acute lung injury, accompanied by robust inflammatory responses termed ‘cytokine storm’.^8,45,46^ Accumulating evidence indicates that aggressive inflammatory cytokine storm is strongly implicated in severe COVID-19 pathophysiology, such as airways damage, ARDS with multiorgan failure, and blood clots.^1,8,9,47^ The severity of COVID-19 is associated with the load of SARS-CoV-2, as well as the activation of host immune responses, especially inflammatory responses.^9^ Therefore, restricting dysfunctional inflammatory responses is a critical step during anti-SARS-CoV-2 therapies.^1,9^ However, the molecular mechanism by which SARS-CoV-2 triggers dysfunctional inflammatory responses remains elusive. In this study, we showed SARS-CoV-2 infection led to the induction of inflammatory responses and the release of multiple cytokines, like IL-6, IL-1β, TNFα, and CXCL10, through promoting the activation of NF-κB signaling pathway. In addition, SARS-CoV-2 N protein promoted NF-κB signaling and induced inflammatory responses (Fig. 5h).

Several other viral proteins of SARS-CoV-2 are recently shown to encounter host innate immunity. SARS-CoV-2 nsp1 protein could reduce immune responses by inhibiting translation.^48–50^ SARS-CoV-2 nsp3 attenuates type I IFN responses by cleaving ISG15 from IRF3.^51^ Our recent study elaborated that SARS-CoV-2 nsp5 could impair both virus-triggered type I IFN production and the downstream ISG induction.^52^ Membrane (M) protein inhibits type I and III IFN production by targeting RIG-I/MDA-5.^53^ ORF6 is shown to inhibit both type I IFN production and downstream signaling.^54,55^ Despite most of the recent identified SARS-CoV-2 viral proteins are shown to restrict host immune response, which is regarded as a common defense strategy that pathogenic viruses use to replicate and propagate in their host, our study revealed the SARS-CoV-2 N protein could positively regulate inflammatory responses through promoting NF-κB activation. Unlike other viruses, SARS-CoV-2 was also shown to induce robust inflammatory responses at a later stage of infection, which led to an inflammatory disease state associated with COVID-19 and even inflammatory cytokine storm.^1,4^ However, the detailed mechanisms of SARS-CoV-2 to induce inflammatory responses remains unclear.^9^ Thus, our study here may partially provide an explanation about how SARS-CoV-2 induces severe inflammatory responses.

Based on these results, we proposed the working model of SARS-CoV-2 which suppresses host innate immunity by its proteins for immune evasion at an early stage of infection, while it induces severe inflammatory responses through N protein at later stage of infection. It has been reported that SARS-CoV-2 could generate a set of discontinuous viral RNA called sub-genomic RNA (sgRNA) during viral replication and transcription, which may lead to the proportion change of different viral proteins.^2,56^ These results might explain why SARS-CoV-2 could induce excessive inflammation during viral assemble. Consistent with our working model, recent clinical research proposed a “two-stage” pattern of disease progression and mechanism of pathogenesis during SARS-CoV-2 infection.^57^ This model indicated that at early stage of COVID-19 disease, the immune system is suppressed due to the inhibitory effect of a number of viral proteins on the immune signaling pathway. Late stage of infection shows an activated immune response to some extent, leading to cytokine storm syndrome at the severe COVID-19 patients.^57^ Our study here provided the molecular mechanism of the enhanced immune response by viral infection at a late stage in their model.

LLPS has emerged as a fundamental cellular mechanism underlying the formation of membraneless organelles, which can concentrate or compartmentalize cellular reactions.^58,59^ Previous research showed that N protein of SARS-CoV could form oligomerization and resemble condensates in cells,^37^ suggesting the LLPS capability of SARS-CoV N protein. Several recent and not-yet peer-reviewed studies revealed that viral RNA binds to N protein of SARS-CoV-2 and induces a robust phase transition to liquid-like condensate.^39–43,60,61^ Several studies showed that N protein/RNA phase separation may promote viral assembly by triggering the aggregation of viral genome and N protein.^39–41,61^ Other study showed that N protein/RNA phase separation promoted RNA synthesis by recruiting viral RNA polymerase.^43^ It is likely that inhibition of N protein phase separation could affect assembly and replication of SARS-CoV-2.^61,62^ However, the involvement of LLPS during SARS-CoV-2-induced immune responses remains largely unclear. Here we found N protein/RNA droplets offer a platform to maximize the interaction between IKKβ and TAK1 in which TAK1 fully co-localized with N protein, while IKKβ surrounding the TAK1/N/RNA droplets. This pattern was not only observed in vitro, but also discovered in vivo. We hypothesized that TAK1/N/RNA form the “activation core” to recruit and activate downstream molecules such as IKKβ. Once IKKβ were recruited and activated by TAK1, it would drift away to form the IKK complex and activate downstream molecules. The inhibition of N protein-mediated LLPS by 1,6-HEX dissolved liquid-like assemblies of N protein, which could reduce the association between TAK1 and IKKβ as well as the activation of NF-κB signaling.

The ongoing COVID-19 pandemic demands novel and effective therapies to restrict viral infection and lessen disease severity. Restraining the dysfunctional inflammatory response may be equally important as the clearance of the virus. Our finding expounded that N protein/RNA phase separation enhances the association between TAK1 and IKK complex and promotes NF-κB-dependent inflammatory responses. Considering the pivotal roles of N protein in the viral life cycle, disrupting the N protein condensates not only interrupts life cycle of SARS-CoV-2, but also reduces SARS-CoV-2-induced inflammatory responses. Such a strategy may provide an important complement for potential therapeutic intervention on SARS-CoV-2 infection.

## Materials and methods

### Cell lines

Vero E6 cells, HEK293T cells, HeLa cells, A549 cells, and Huh7 cells were cultured in DMEM (Corning) with 10% (vol/vol) fetal bovine serum (Gibco) in a 5% CO_2_ incubator at 37 °C. Calu3 cells were cultured in MEM (Gibco) with 1% NEAA (Gibco), 1% SODIUM PYRUVATE (Gibco) and 10% (vol/vol) fetal bovine serum (Gibco) in a 5% CO_2_ incubator at 37 °C.

### Viruses

As previously described,^63^ Vero E6 cells were used to generate SARS-CoV-2 (Accession number: MT123290) virus and measure the titer of SARS-CoV-2 virus. SARS-CoV-2 virus was adsorbed in 2 h with gentle rocking every 15 min before incubation at 37 °C for indicated time. Cells were infected at different MOI and time points as indicated.

### Antibodies and reagents

Horseradish peroxidase (HRP)-anti-Flag (M2, A8592) and anti-β-actin (A1978) were purchased from Sigma. Anti-hemagglutinin-HRP (HA, 12013819001) and mouse monoclonal anti-c-Myc-HRP (11814150001) were purchased from Roche Applied Science. Anti-p-IKKα/β (2697), anti-IκBα (4814), anti-p-IκBα (9246), and anti-p65 (59674), anti-p-p65 (3033), anti-p38 MAPK (9212), anti-p-p38 MAPK (9211), anti-JNK (9252), anti-p-JNK (9251), anti-Erk1/2 (9102), and anti-p-Erk1/2 (9101) were acquired from Cell Signaling Technology. Anti-TAK1 (YT4536) was acquired from ImmunoWay. Anti-N (40588-T62) was purchased from Sino Biological. Anti-IKKα (14A231, NB100-56704) was purchased from NOVUS. Anti-IKKβ (05-535) was purchased from Millipore. Dsuccinimidyl suberate (DSS) (21655) was purchased from Thermo Scientfic. poly(I:C) (LMW) was purchased from Invivogen. polyU (P9528), Doxycycline (D9891), Digitonin (D141), and 1,6-hexanediol (230117) were acquired from Sigma. TPCA-1 (HY-10074), BAY 11-7082 (HY-13453), JSH-23 (HY-13982), and T6167923 (HY-19744) were acquired from MedChemExpress (MCE).

### Luciferase and reporter assays

293T (2 × 10^5^) cells were plated in 24-well plates and transfected with NF-κB-luciferase (luc) reporter plasmid (firefly luciferase, 50 ng), pRL-TK plasmid (renilla luciferase, 10 ng) and poly(I:C) (1 µg/ml) or plasmid encoding MyD88, IRAK1, TRIF, RIPK1, TRAF2, TRAF6, TAK1, TAB1, IKKβ, or p65 together with indicated variety expression plasmid of viral proteins or empty vector (pcDNA3.1) plasmid using Lipofectamine 2000 (Invitrogen). Cells were collected 48 h later and measured with the Dual-Luciferase Assay (Promega) with a Luminoskan Ascent luminometer (Thermo Scientific). Data represent relative firefly luciferase activity normalized to renilla luciferase activity.

### Plasmids

Plasmids encoding SARS-CoV-2 proteins were chemically synthesized based on SARS-CoV-2, as previously described.^52^ We sub-cloned these viral proteins into pcDNA3.1 expression vector using standard PCR techniques. NF-κB-luciferase reporter plasmid has been described previously.^64^ TAK1, TAB1, IKKα, and IKKβ were also acquired by the means of standard PCR techniques and cloned into pcDNA3.1-HA, pcDNA3.1-Flag, or pcDNA3.1-Myc vectors. TAK1, IKKβ, SARS-CoV-2 N protein (FL), N-△NTD (delete amino acid residues 44-180), N-△CTD (delete amino acid residues 247-364), and N-△SR (delete amino acid residues 181-246) conjugated Flag tag were subsequently cloned into pEGFP-C2 or pmCherry-C1 vectors. pET-28a (+) vector was used to construct the expressing plasmids encoding N protein, TAK1, and IKKβ conjugated EGFP or mCherry tag to purify the proteins in vitro.

### Generation of expressing cell lines

For protein N-inducible and N-stable expression, lentiviral particles were produced respectively by transfecting HEK293T cells with FG-EH-DEST-N-Puro vector or N-lentiviral transfer vector, together with pLP1, pLP2, and Plp/VSVG vectors. A549 cells expressing Teton-3G were infected by lentivirus-containing supernatant for N-inducible cells. Huh7, Calu3, and HeLa cells were used to construct the N-stable expressing cell lines. All cell lines were selected to 3 µg/ml puromycin twice as previously described.^52,65^

### Protein expression and purification

The expressing plasmids encoding N protein, TAK1, and IKKβ conjugated EGFP or mCherry tag were transformed into *E.coli* BL21. Freshly transformed cells were grown in LB containing Kanamycin (50 µg/ml) to OD600 0.6 at 37 °C for about 12 h. After induction with 1 mM IPTG at 37 °C for 6 h, cells were harvested by centrifugation at 4000 rpm at 4 °C for 10 min and were resuspended in His-lysis buffer (50 mM Tris-HCl pH 7.8, 150 mM NaCl, 1% Triton X-100, 2 mM DTT [Sigma, 10197777001], 20 mM imidazole [Sigma, 56750], and protease inhibitors [Roche, 11697498001]). Cells were lysed by sonication on ice and centrifuged (12,000 rpm, 30 min, 4 °C) to remove debris. The supernatant was purified by incubation with Ni-NTA agarose beads (QIAGEN, 30210) overnight at 4 °C. Ni-NTA beads were then washed with wash buffer (50 mM NaH_2_PO_4_, 300 mM NaCl, 20 mM imidazole, pH 7.8), and proteins were eluted with elution buffer (50 mM NaH_2_PO_4_, 300 mM NaCl, 300 mM imidazole, pH 7.8). The purified proteins were further dialyzed by using PD10 column (GE Healthcare), followed by concentrating to 100–300 μL using Amicon Ultra 30 K (Millipore, C134281) concentrators at 4 °C, then quantified by the BCA method (Pierce, 23250) and stored at −80 °C. After purification, all the proteins were quantified by A260/A280 (below 0.55) measurement to guarantee the exclusion of nucleic acid.

### RNA extraction and quantitative RT-PCR

Total RNA was extracted from cells using Trizol reagent (Invitrogen) according to the manufacturer’s instructions. cDNA was generated with HiScript® III RT SuperMix for qPCR (+gDNA wiper) (Vazyme, R323-01) and was analyzed by quantitative real-time PCR using the 2× RealStar Green Power Mixture (GenStar, A311-01). RT-PCR was performed in a Roche LightCycler 480 System (Basel, Switzerland). All data were normalized to RPL13A expression. Primer sequences were listed below:

*RPL13A*: Forward: 5′-GCCATCGTGGCTAAACAGGTA-3′

Reverse: 5′-GTTGGTGTTCATCCGCTTGC-3′

*IL-1B*: Forward: 5′-AGCTACGAATCTCCGACCAC-3′

Reverse: 5′-CGTTATCCCATGTGTCGAAGAA-3′

*IL-6*: Forward: 5′-ACTCACCTCTTCAGAACGAATTG-3′

Reverse: 5′-CCATCTTTGGAAGGTTCAGGTTG-3′

*TNF*: Forward: 5′-CCTCTCTCTAATCAGCCCTCTG-3′

Reverse: 5′-GAGGACCTGGGAGTAGATGAG-3′

*CXCL2*: Forward: 5′-CTTGCCAGCTCTCCTCCTC-3′

Reverse: 5′-AGGGGCGCTCCTGCT-3′

*CXCL8*: Forward: 5′-ACTGAGAGTGATTGAGAGTGGAC-3′

Reverse: 5′-AACCCTCTGCACCCAGTTTTC-3′

*CXCL10*: Forward: 5′-GTGGCATTCAAGGAGTAGCTC-3′

Reverse: 5′-GCCTTCGATTCTTGGATTCAG-3′

*CCR1*: Forward: 5′-GACTATGACACGACCACAGAGT-3′

Reverse: 5′-CCAACCAGGCCAATGACAAATA-3′

### Immunoprecipitation and immunoblot analysis

For immunoprecipitation, whole-cell extracts were prepared after infection, transfection, or stimulation with appropriate ligands, followed by incubation overnight with anti-Flag or anti-HA agarose gels (Sigma) at 4 °C. Beads were then washed 3–5 times with low-salt lysis buffer (50 mM HEPES, 150 mM NaCl, 1 mM EDTA, 10% glycerol, 1.5 mM MgCl_2_, and 1% Triton X-100), and immunoprecipitates were eluted with 2× SDS Loading Buffer and resolved by SDS-PAGE. Then proteins were transferred to PVDF membranes (Bio-Rad) and further incubated with the appropriate antibodies. Immobilon Western Chemiluminescent HRP Substrate (Millipore, WBKLS0500) was used for protein detection.

### Fluorescence microscopy

Cells were cultured on Glass Bottom culture dishes (Nest Scientific). After stimulation, cells were fixed by 4% paraformaldehyde for 10 min and washed with cold PBS for three times before permeabilized with methyl alcohol for 30 min at −20 °C. After washing with cold PBS for three times, cells were blocked in 6% goat serum (Boster Biological, AR1009) for 1 h at room temperature, and then incubated with primary antibodies diluted in 6% goat serum overnight. Cells were washed with PBS for three times and subsequently incubated with fluorescently labeled secondary antibodies (Alexa Fluor 488- and Alexa Fluor 568-conjugated antibodies against mouse, rabbit) for 1 h. Confocal images were examined using a microscope (TCS SP8 STED 3X, Leica) equipped with 100 × 1.40 NA oil objectives. The images were processed for gamma adjustments using Leica AS Lite or ImageJ software (National Institutes of Health).

### Fluorescence recovery after photobleaching (FRAP)

FRAP assay was conducted using the FRAP module of the Leica SP8 confocal microscopy system. The EGFP-N or mCherry-N was bleached using 488-nm or 568-nm laser beam separately. Bleaching was focused on a circular region of interest (ROI) using 100% laser power and time-lapse images were collected. Fluorescence intensity was measured and normalized relative to pre-bleaching time points by Leica AS Lite software. GraphPad Prism 8 is used to plot and analyze the FRAP results.

### In vitro LLPS assay

For the LLPS assay, the purified proteins were mixed with 1 × PBS and 2.5% PEG8000 (Sigma), followed by incubating with poly(I:C) or polyU at the indicated concentration for 15 min at 25–37 °C. Ten microliters of each sample were pipetted onto a Glass Bottom culture dish and imaged using a Leica microscope (TCS SP8 STED 3X, Leica). To acquire a higher solution image of the droplet, we picked up the highlighted area and used 5× confocal zoom-in method to re-photograph it. The fluidity of the droplet may lead to different outputs between overview images and the corresponding enlarged images.

### RNA sequencing

Total RNA was isolated from cells using TRIzol reagent (Invitrogen), and sequencing was performed by Sangon Biotech. Sample quality was assessed using a Bioanalyzer (Agilent 2100 Bioanalyzer). RNA-seq libraries of polyadenylated RNA were prepared using mRNA-seq V2 Library Prep Kit and sequenced on MGISEQ-2000 platform. All clean data were mapped to the human genome GRCh38 using HISAT2 v2.1.0 with default parameters. Bam files were sorted by Samtools 1.9. Reads counts were summarized using the featureCounts program^66^ as part of the Subread package release 2.0.0 (http://subread.sourceforge.net/). To identify DEGs between groups, DESeq2 was used to normalize reads counts and *p* value < 0.05 and absolute logged fold-change ≥ 1 were determined as DEGs^67^ using Bioconductor clusterProfiler package v3.14.3 for the functional enrichment of DEGs.^68^ RNA-seq sequence density profiles were normalized using bamCoverage and visualized in IGV genome browser.^69^

### Statistical analyses

Images were analyzed with ImageJ. All data are presented as mean ± SEM unless from independent determinations, and statistical analyses were done using the software Graphpad Prism (GraphPad Software, Inc, La Jolla, CA, USA). Differences of means were tested for statistical significance with unpaired two-tailed Student’s *t* test. *p < 0.05; **p < 0.01; ns, not significant.

## Supplementary information

Supplemental Material

## Data Availability

All data supporting the findings of this study are available within the article and its supplementary information or from the corresponding author upon reasonable request. Source data are provided with this paper.
