# Supplementary material for: RNA-induced liquid phase separation of SARS-CoV-2 nucleocapsid protein facilitates NF-κB hyper-activation and inflammation
Source: Signal Transduct Target Ther. 2021 Apr 24;6:167. doi: 10.1038/s41392-021-00575-7 (PMC8065320; doi:10.1038/s41392-021-00575-7)
Supplement: Supplementary file 1 — Supplemental Material [file 41392_2021_575_MOESM1_ESM.docx]

Supplementary Materials for

**RNA-induced Liquid Phase Separation of SARS-CoV-2 Nucleocapsid Protein Facilitates NF-κB Hyper-activation** **and Inflammation**

Yaoxing Wu, Ling Ma, Sihui Cai, Zhen Zhuang, Zhiyao Zhao, Shouheng Jin, Weihong Xie, Lingli Zhou, Lei Zhang, Jincun Zhao and Jun Cui

Correspondence to: Jun Cui (cuij5@mail.sysu.edu.cn) or Jincun Zhao (zhaojincun@gird.cn).

**This PDF file includes:**

**Figures. S1 to S6**

Figure. S1.


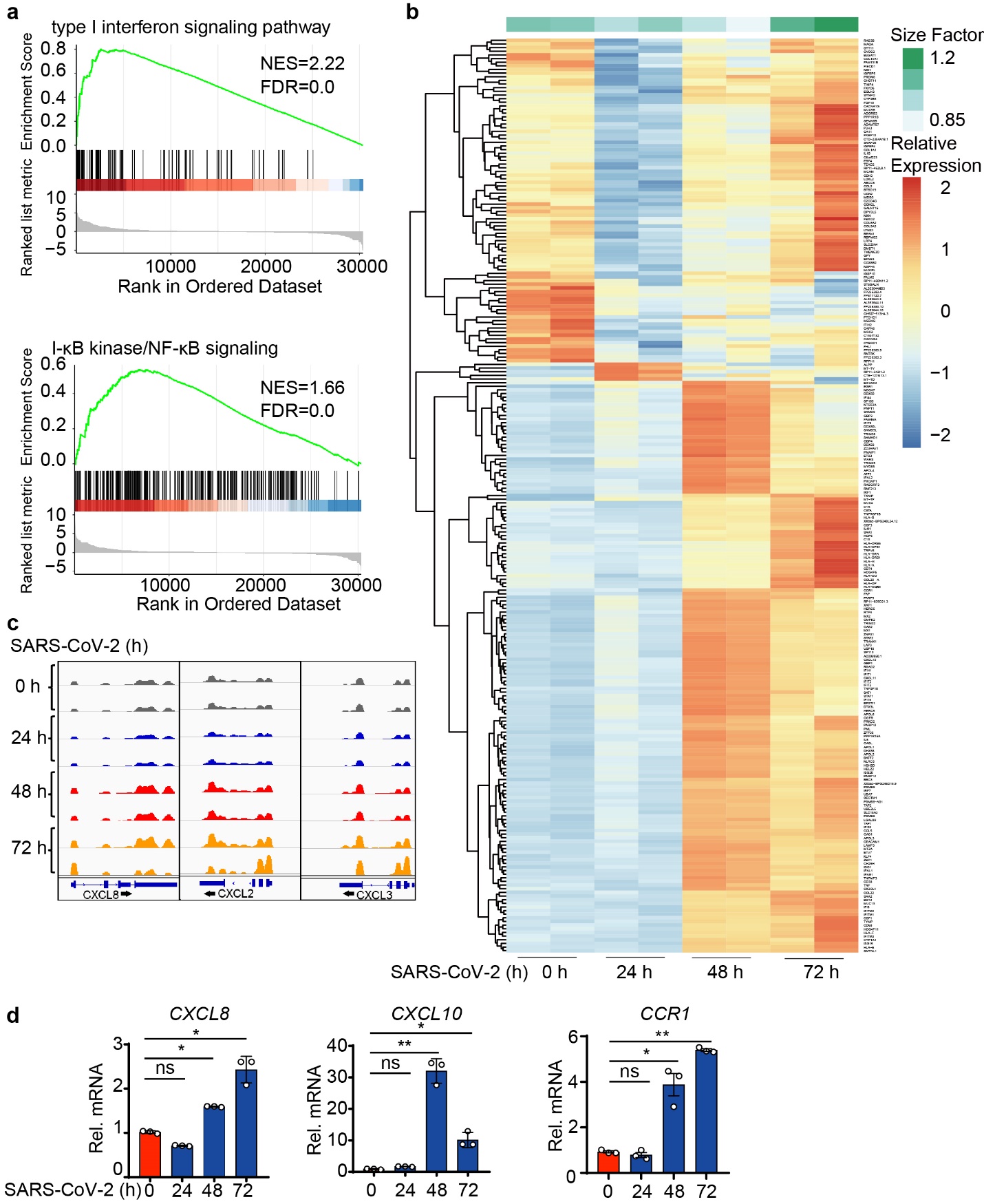


**Figure. S1. SARS-CoV-2 infection induces the activation of host NF-κB signaling and inflammatory responses.** **a** GSEA analysis of genes expressed differentially in SARS-CoV-2-infected Calu3 cells at 48 hours post infection. FDR (q-value) was shown. **b** Top 100 principal upregulated DEGs (p-value < 0.05; log2(FC)＞1) in SARS-CoV-2-infected Calu3 cells at indicated time points post infection. **c** IGV browser tracks showing the RNA-seq signals of inflammatory response genes in Calu3 cells at indicated time points post infection. **d** Quantitative PCR with reverse transcription analysis of NF-κB relative cytokines genes (*CXCL8, CXCL10, CCR1*) of Calu3 cells with SARS-CoV-2 infection at MOI=0.05 for indicated time points. Data in (**d**) are expressed as mean ± SEM of at least three independent experiments. *p < 0.05, **p < 0.01, ns, not significant (two-tailed Student’s *t*-test).

Figure. S2.


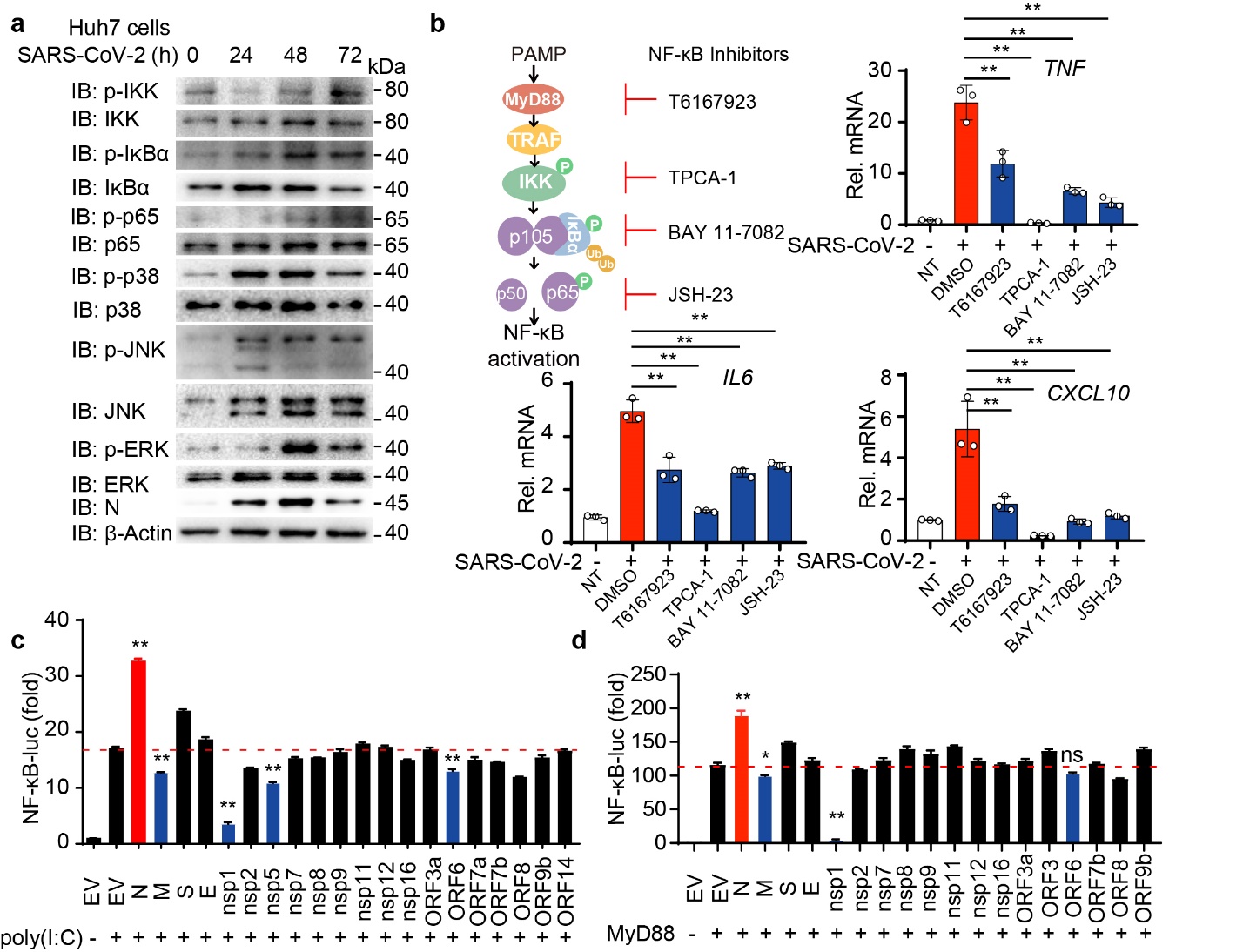


**Figure. S2. SARS-CoV-2 enhances NF-κB signaling. a** Lysates of Huh7 cells infected with SARS-CoV-2 at MOI=0.2 for indicated time points were collected and immunoblotted with indicated antibodies. **b** Schematic overview (left) of NF-κB signaling pathway and the target of its inhibitors. Quantitative PCR (right and below) with reverse transcription analysis of *TNF*, *IL6* and *CXCL10* mRNA in Calu3 cells with SARS-CoV-2 infection at MOI=0.05 for 24 hours with DMSO, T6167923 (20 μM), TPCA-1 (5 μM), BAY 11-7082 (20 μM) and JSH-23 (10 μM) treatment for 24 hours before harvested. **c-d,** HEK293T cells were transiently transfected with NF-κB luciferase reporter, TK-luc along with empty vector (EV) or proteins of SARS-CoV-2, followed by 1 μg/mL poly(I:C) (**c**) treatment for 12 hours or with MyD88 (**d**) overexpression for 24 hours. Lysates were collected 36 hours post-transfection and luciferase activities were tested. Data in (**b-d**) are expressed as mean ± SEM of at least three independent experiments. **p < 0.01, ns, not significant (two-tailed Student’s *t*-test). For **a**, similar results are obtained for three independent biological experiments.

Figure. S3.


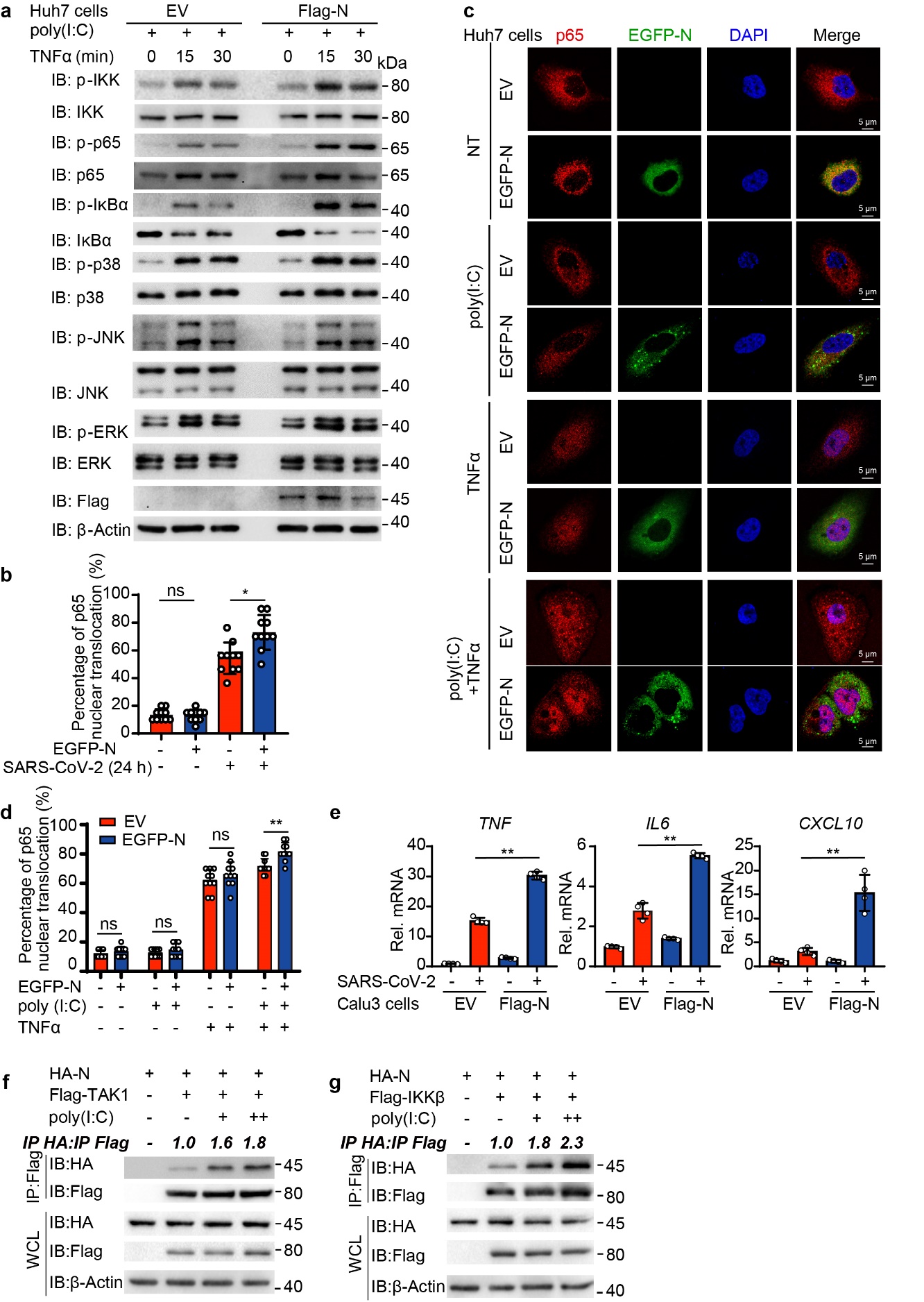


**Figure. S3. N protein of SARS-CoV-2 promotes NF-κB signaling pathway through TAK1 and IKKβ. a** Huh7 cells stably expressing EGFP-N protein or EV were treated with 1 μg/mL poly(I:C) for 6 hours and 50 ng/mL TNFα for indicated time points. Cell lysates were harvest for immunoblotting with indicated antibodies. **b** Quantitative analysis of the percentage of p65 nuclear translocation in cells of **Fig. 1h** (10 cells per sample). **c** EGFP-N protein or EV stably expressing-Huh7 cells were treated with 1 μg/mL poly(I:C) for 6 hours and 50 ng/mL TNFα for 2 hours or left non-treated (NT), followed by labeling p65 and DAPI. Confocal microscopy of p65 localization was performed. Scale bars, 5 μm. **d** Quantitative analysis of the percentage of p65 nuclear translocation in Huh7 cells of **Supplementary** **Fig. S3c** (10 cells per sample). **e** Quantitative PCR with reverse transcription analysis of *TNF*, *IL6*, *CXCL10* in Calu3 cells stably expressing N protein with SARS-CoV-2 infection at MOI=0.05 for 24 hours. **f-g** HEK293T cells were transiently transfected with HA-N, Flag-TAK1 **(f)** or Flag-IKKβ **(g)** and treated with 1 μg/mL poly(I:C) for 6 hours. Cell lysates were collected for co-IP with anti-Flag beads and immunoblotted with indicated antibodies. The ratio of the gray value between IP-HA and IP-Flag were determined. Data in **(e)** are expressed as mean ± SEM of at least three independent experiments. Data in **(b)** and **(d)** are expressed as mean ± SD. **p < 0.01, ns, not significant (two-tailed Student’s *t*-test). For **(a) (c)** and **(f-g)** similar results are obtained for three independent biological experiments.

Figure. S4.

**
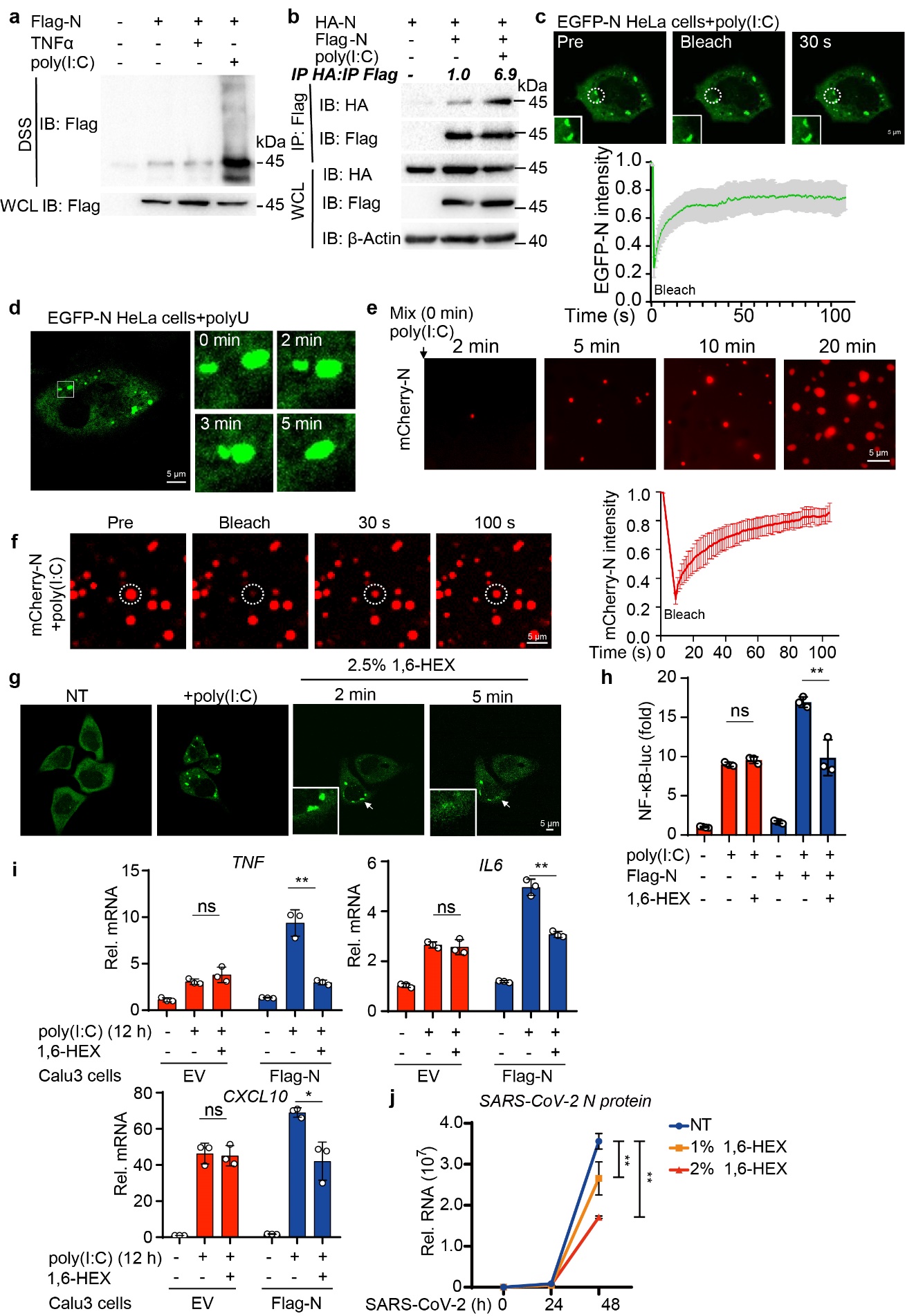
**

**Figure. S4. SARS-CoV-2 N protein undergoes LLPS to promote NF-κB signaling. a** HEK293T cells were transfected with Flag- EV and Flag- SARS-CoV-2 N plasmid, treated with 50 ng/mL TNFα for 30 min or 1 μg/mL poly(I:C) for 6 hours. Cell lysates were collected and treated with DSS for immunoblotting with indicated antibodies. **b** HEK293T cells were transfected with HA-N, Flag-EV and Flag-N, followed by 1 μg/mL poly(I:C) treatment for 6 hours. Cell lysates were collected for co-IP with anti-Flag beads and immunoblotting with indicated antibodies. The ratio of the gray value between IP-HA and IP-Flag were determined. **c** Representative images (left) of FRAP assay of N protein-poly(I:C) droplets in HeLa cells. HeLa cells stably expressing EGFP-N protein were treated with 1 μg/mL poly(I:C). Bleaching was performed at the indicated time points and the recovery occurred at 37 °C. Scale bars, 5 μm. Fluorescence intensity analysis (right) of fluorescence recovery after photobleaching over a 120-seconds time course. **d** Representative images of fusion of two EGFP-N protein-polyU puncta in HeLa cells stably expressing EGFP-N protein, along with 1 μg/mL polyU treatment for 6 hours. Scale bar, 5 μm. **e** Representative images of time-lapse imaging of N protein-poly(I:C) phase separation *in vitro*. Liquid droplets were formed after mixing of 10 μM mCherry-N protein and 10 ng/mL poly(I:C) and matured at 37 °C for 15 minutes. **f** Representative images (left) of FRAP assay of mCherry-N protein-poly(I:C) droplets *in vitro*. 10 μM mCherry-N protein mixed with 10 ng/mL poly(I:C) and incubated at 37 °C for 15 minutes. Bleaching was performed at the indicated time points and the recovery occurred at 37 °C. Scale bar, 5 μm. Fluorescence intensity analysis (right) of fluorescence recovery after photobleaching over a 120-seconds time course. **g** Representative images of time-lapse micrographs of HeLa cells stably expressing EGFP-N protein with 1 μg/mL poly(I:C) treatment for 6 hours, together with 20 μg/mL digitonin and 2.5% 1.6-hexanediol treatment for indicated time points. **h** HEK293T cells were transfected with NF-κB luciferase reporter, TK-luc and Flag-EV or Flag-N, followed by 1 μg/mL poly(I:C) treatment for 12 hours, along with 20 μg/mL digitonin and 2.5% 1.6-hexanediol treatment for 2 hours before harvested. Cell culture supernatants were collected at 36 hours post-transfection and luciferase activities were tested. **i,** Quantitative PCR with reverse transcription analysis of *TNF*, *IL6*, and *CXCL10* in Calu3 cells stably expressing SARS-CoV-2 N protein with 1 μg/mL poly(I:C) treatment for 12 hours, along with 20 μg/mL digitonin and 2.5% 1.6-hexanediol treatment for 2 hours before harvested. **j** Quantitative PCR with reverse transcription analysis of *SARS-CoV-2 N protein* of Calu3 cells with SARS-CoV-2 infection at MOI=0.05 for indicated time points and followed by treatment of 20 μg/mL digitonin and different doses of 1,6-HEX for 2 hours. Data in **(c) (f) (h) (i)** and **(j)** are expressed as mean ± SEM of at least three independent experiments. *p < 0.05, **p < 0.01, ns, not significant (two-tailed Student’s *t*-test for c, f, h and i or two-way ANOVA for j). For **(a-g)**, similar results are obtained for three independent biological experiments.

Figure. S5.


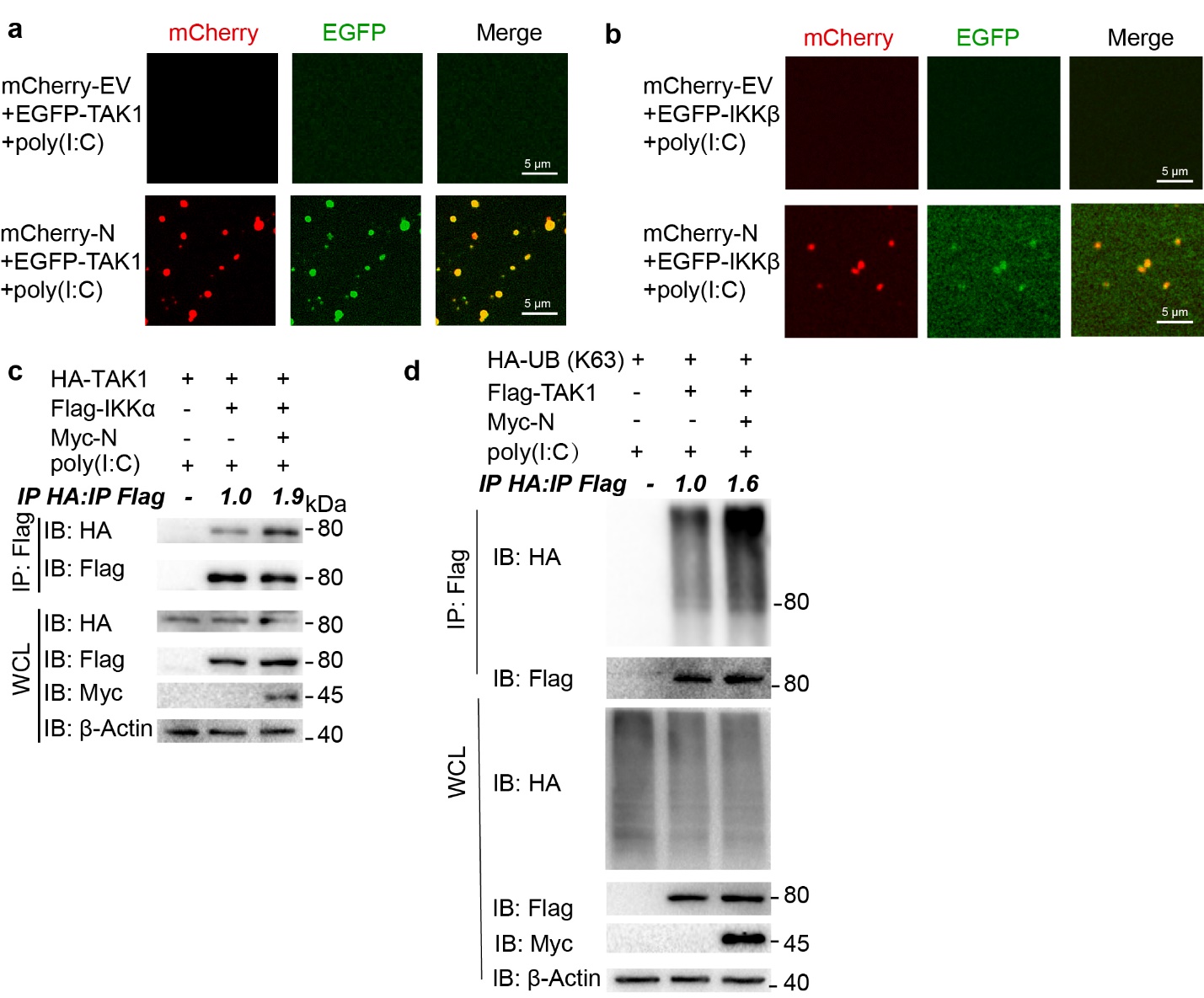


**Figure. S5. LLPS of SARS-CoV-2 N protein recruits TAK1 and IKK complex. a-b** Representative images showing TAK1 **(a)** or IKKβ **(b)** were distributed into N protein-poly(I:C) droplets. 10 µM of mCherry-N protein, 5 µM of EGFP-TAK1 or EGFP-IKKβ were mixed with 10 ng/ml of poly(I:C) and incubated at 37 °C for 15 minutes. Scale bars, 5 μm. **c** HEK293T cells were transfected with indicated plasmids combinations, followed by 1 μg/mL poly(I:C) treatment for 6 hours. Cell lysates were harvested for co-IP with anti-Flag beads and immunoblotting with indicated antibodies. **d** HEK293T cells were transfected with HA tagged ubiquitin HA-UB (K63), Myc-N and Flag-TAK1 for 1 μg/mL poly(I:C) treatment for 6 hours. Cell lysates were harvested for co-IP with anti-Flag beads and immunoblotting with indicated antibodies. For **(a-d)**, similar results are obtained for three independent biological experiments.

Figure. S6.


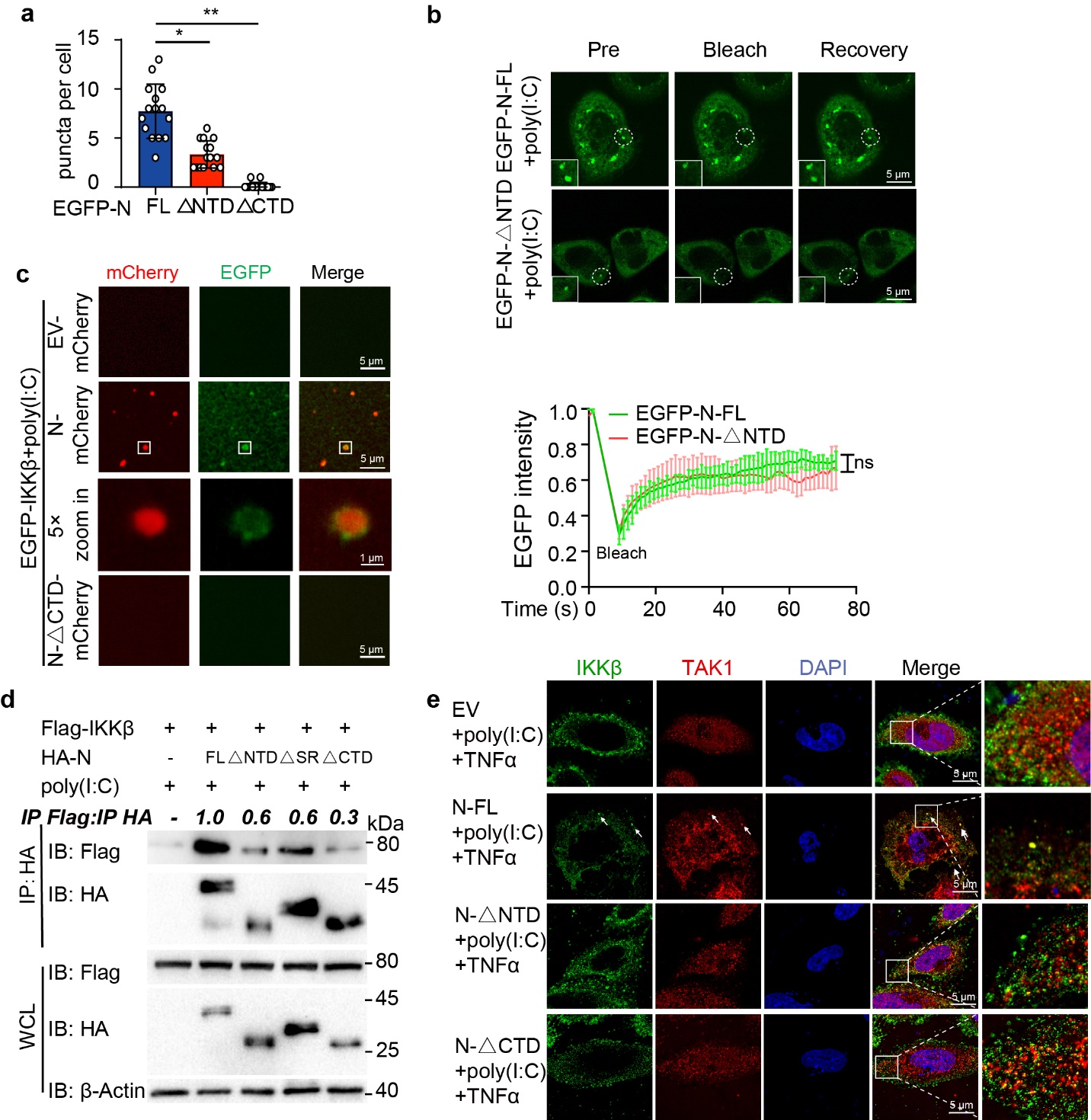


**Figure. S6. CTD domain of SARS-CoV-2 N protein is critical for its LLPS and NF-κB regulatory capability.** **a** The quantitative analysis of the number of droplets of **Fig. 5c** (15 cells each sample). **b** Representative images (up) of FRAP assay in HeLa cells stably expressing EGFP-N protein or EGFP-△NTD with 1 μg/mL poly(I:C) treatment. Bleaching was performed and the recovery occurred at 37 °C. Scale bars, 5 μm. Representative images (down) of FRAP assay of N protein-poly(I:C) droplets in HeLa cells. **c** Representative images showing IKKβ was distributed into N protein-poly(I:C) droplets. 10 μM of mCherry-N protein or mCherry-N protein-△CTD, 5 μM of EGFP-IKKβ were mixed with 10 ng/ml of poly(I:C) and incubated at 37 °C for 10 minutes. Confocal 5× zoom-in of the area highlighted above was re-photographed. Scale bars, 5 μm. **d** HEK293T cells transfected with Flag-IKKβ, HA-EV, HA-N FL, △NTD, △SR and △CTD, followed by 1 μg/mL poly(I:C) treatment for 6 hours, were harvested for co-IP with anti-HA beads and immunoblotting with indicated antibodies. The ratio of the gray value between IP-Flag and IP-HA were determined. **e** Confocal microscopy of colocalization between IKKβ and TAK1 in HeLa cells expressing N FL, △NTD and △CTD, followed by 1 μg/mL poly(I:C) treatment and 50 ng/mL TNFα treatment. Scale bars, 5 μm. Data in **(a-b)** are expressed as mean ± SD. *p < 0.05, **p < 0.01, ns, not significant (two-tailed Student’s *t*-test for a and two-way ANOVA for b). For **(b-e)**, similar results are obtained for three independent biological experiments.
